# 

**Published:** 1888-08-11

**Authors:** 


					'PRICE ONE PENNY. ?
Per Annnin. fis. fld.. nnut free.
i, Vol. XV.-
-August 11th, 1888.
Per Annum, 6s. 6d? post free.
Monthly Farts, 6d.; post free, 7id.
Ditto per Ann., 7s. 6d. post free.
\r_< ?  ? - ?\ ?i MP51 jaonmiy raris, ea.; past iree. /ta,
l?egisicre?l at the Qeneral Post Oillce W^1 ? ?
as a Newspaper.J Ditto per Ann., 7s. 0d. post free.

				

